# Health Literacy Measure for Adolescents (HELMA): Development and Psychometric Properties

**DOI:** 10.1371/journal.pone.0149202

**Published:** 2016-02-16

**Authors:** Shahla Ghanbari, Ali Ramezankhani, Ali Montazeri, Yadollah Mehrabi

**Affiliations:** 1 Department of Public Health, School of Health, Shahid Beheshti University of Medical Sciences, Tehran, Iran; 2 Mental Health Research Group, Health Metrics Research Centre, Iranian Institute for Health Sciences Research, ACECR, Tehran, Iran; 3 Department of Epidemiology, School of Health, Shahid Beheshti University of Medical Sciences, Tehran, Iran; University Of São Paulo, BRAZIL

## Abstract

**Background:**

Health literacy refers to personal competencies for the access to, understanding of, appraisal of and application of health information in order to make sound decisions in everyday life. The aim of this study was to develop and evaluate the psychometric properties of an instrument for the measurement of health literacy among adolescents (the Health Literacy Measure for Adolescents-HELMA).

**Methods:**

This study was made up of two phases, qualitative and quantitative, which were carried out in 2012–2014 in Tehran, Iran. In the qualitative part of the study, in-depth interviews with 67 adolescents aged 15–18 were carried out in 4 high schools to generate the initial item pool for the survey. The content validity of the items was then assessed by an expert panel review (n = 13) and face validity was assessed by interviewing adolescents (n = 16). In the quantitative part of the study, in order to describe the psychometric properties of the scale, validity, reliability (internal consistency and test-retest) and factor analysis were assessed.

**Results:**

An item pool made up of 104 items was generated at the qualitative stage. After content validity was considered, this decreased to 47 items. In the quantitative stage, 582 adolescents aged 15–18 participated in the study with a mean age of 16.2 years. 51.2% of participants were females. In principal component factor analysis, 8 factors were loaded, which accounted for 53.37% of the variance observed. Reliability has been approved by α = 0.93 and the test-retest of the scale at two-week intervals indicated an appropriate stability for the scale (ICC = 0.93). The final questionnaire was approved with 44 items split into eight sections. The sections were titled: gain access to, reading, understanding, appraise, use, communication, self-efficacy and numeracy.

**Conclusion:**

The Health Literacy Measure for Adolescents (HELMA) is a valid and reliable tool for the measurement of the health literacy of adolescents aged 15–18 and can be used to evaluate different levels of functional, interactive, and critical health literacy in adolescents.

## Introduction

Health literacy was introduced around 40 years ago [1,2] and since then has become an issue considered to be important in the public’s health and in recent years, it has received substantial attention [3,4].

Health literacy was defined from different perspectives and thus various definitions have been given to it [1,5,6]. Recently, the European Consortium on Health Literacy Score (HLS) gave a more comprehensive definition of health literacy. They suggested four key factors for health literacy: the ability to access, understand, appraise, and apply health information in order to make decisions in everyday life in the three main areas of disease prevention, healthcare, and health promotion [2,4,6]. Most studies on health literacy took place in medical settings and showed that lower health literacy was associated with poorer health outcomes, health disparities, and increased healthcare expenditure [7–10].

Health literacy is widely recognized as the most important social determinant of health and outcome of health education endeavors [6,11,12]. Furthermore, it includes competencies such as interactive and critical skills that are necessary to better control one's health [13]. In public health, health literacy is assumed to be an asset that can reduce health disparities [14] and improve social capital [12]. It brings together the fields of education and health and creates the framework to enhance individuals' abilities to achieve good health throughout their life [15,16].

Adolescence is an important period of life, with specific regard to independent decision-making. It is therefore a crucial time to arm adolescents with accurate and reliable health information so they can develop lifelong healthy behavior [17], as well as to enable them to take control of the condition of their own health [18].

Broad access to information via the Internet has given rise to great concerns about adolescent health literacy and the validity of the information they obtain. Limited search abilities, or low literacy skills, make it difficult for adolescents to find and understand good quality information. There is not a school health curriculum, meaning adolescents may not achieve optimal health literacy, which in turn may lead to poorer health outcomes and health disparity [17,19].

Most researchers agreed that health literacy is related to the context, so different settings may require different assessment tools. Despite there being many studies on the development and assessment of instruments used for measuring health literacy in clinical settings [20], few studies have addressed the health literacy of adolescents [21], particularly within schools [22]. This is mainly due to the lack of a comprehensive definition of health literacy and lack of proper assessment tools [3]. As suggested by Nutbeam, a good questionnaire for measuring health literacy should be able to assess a person’s ability to gain access to information from different sources, understand health information, and appropriately apply health information to achieve personal benefit [14].

The Rapid Estimate of Adult Literacy in Medicine (REALM) and the Test of Functional Health Literacy in Adults (TOFHLA) are popular health literacy measurement tools, which were primarily designed and used in clinical settings [13] and subsequently used in studies of adolescent health literacy [23,24]. These measures are currently criticized for their limited focus as they merely assess reading and numeracy skills and do not capture further elements of health literacy such as self-efficacy, communication, and critical thinking [13,25,26]. However, some specific instruments for measuring health literacy among adolescents were found. Wu et al. developed a detailed questionnaire which consisted of 47 items for assessing students’ health literacy in high schools [3]. Their instrument was criticized for being time-consuming, difficult to complete, less applicable to public health surveys, and limited on its outcomes surrounding abilities such as the understanding and evaluation of health information.

Abel et al. designed a short multidimensional tool for measuring health literacy in young adults in the context of everyday life [20]. This was found to be very broad and general, despite considering different dimensions of health literacy.

Massey et al., in a study that contextualized the concept of health literacy among insured adolescents in health centers [27], developed a questionnaire made up of 24 items split into 6 sections for measuring the health literacy of adolescents [13]. The instrument focused on the limited aspects of health literacy among specific sub-groups of adolescents in a healthcare setting, thus making its application very limited. We therefore thought that it is time to develop a more general instrument for measuring health literacy in adolescents in order to facilitate future studies on the topic with a broader application. In particular, the presented study aimed to develop a specific multidimensional measure of health literacy for adolescents aged 15–18 and then provide an evaluation of its psychometric properties. We defined and operationalized health literacy as indicated by adolescents and included the following areas in the survey: gain access to, reading, understanding, appraisal, use, communication and numeracy.

## Materials and Methods

This study had two phases, one of which was qualitative, while the other was quantitative. Descriptions of these are provided as follows:

### Phase 1

#### i) Item generation

Using a qualitative approach, 67 in-depth interviews were conducted with high school students aged 15–18 from November 2012 to February 2013. Interviews were carried out in 4 schools in Tehran, Iran. Participants were selected using purposeful sampling.

During the interviews the adolescents’ were asked about literacy, health, health literacy, awareness of health information resources, their previous experiences in relation to searching for health information, and their use of health information and services.

Sampling continued until no further new data emerged, indicating that the data saturation point was reached. The duration of each interview varied from 25 to 40 minutes. All interviews were digitally recorded having obtained the permission of the participants. After completing each interview, it was listened to carefully several times and transcribed word for word. The data was explored using content analysis and an initial pool of 104 items was generated based on this analysis. After a careful review of the items by the research team, the number of items was reduced to 81. The questionnaire was then scrutinized for its content validity and its face validity.

#### ii) Content validity

The content validity of the questionnaire was carried out using qualitative and quantitative approaches. First, 10 specialists in health education and health promotion, public health, epidemiology, educational planning, and management reviewed the questionnaire to check its grammar, wording, item allocation, and scaling. During the quantitative stage, in order to calculate the Content Validity Ratio (CVR), 13 other specialists were asked to assess each item on a 3-point Likert scale (where 1 = essential, 2 = useful but not essential, 3 = not essential). Then, based on Lawshe’s table [28], items that scored greater than or equal to 0.54 were kept in the scale. Throughout this phase, 27 items were removed. In order to calculate the Content Validity Index (CVI), 10 additional expert panelists were asked to determine the relevance, clarity, and simplicity of each item using a 4-point Likert scale. Usually a same group of experts rate the CVR and the CVI. For more precision we used two separate groups of experts. However, in accordance with Waltz and Baussel [29], items with CVI value greater than or equal to 0.79 were accepted and 7 items that did not meet this criterion were deleted. 47 items had a CVI value of greater than or equal to 0.79.

#### iii) Face validity

The face validity of the scale was determined in two stages. In the qualitative stage, 16 adolescents aged 15–18 (8 boys and 8 girls) were recruited using convenience sampling to determine the ambiguity, relevance and difficulty of each item. At this stage, none of the items were removed but 3 items were changed as a result of the adolescents’ suggestions.

During the quantitative stage, the impact score of each item was calculated (impact score = frequency (%) × importance). For this purpose, another sample of 26 adolescents was asked to score the importance of each item on a 5-point Likert scale. The items with impact scores of 1.5 or above were considered to be satisfactory [30]. Following the content validity and face validity checks, the pre-final version of the instrument (the Health Literacy Measure for Adolescents-HELMA) had 47 items and was ready for the quantitative phase.

### Phase 2

#### i) Design and data collection

Psychometric analysis of the HELMA was performed by way of a cross sectional study with a sample of adolescents aged 15–18 who were studying in high school in Tehran. Participants were recruited using a multi stage sampling method. For sampling, Tehran was divided into to 5 regions: north, south, west, east and center. In each region, a district was selected randomly. In the next stage, two high schools from each district (one boys’ school and one girls’ school) were identified. Then, based on the population of each class, 3 to 4 classes were assigned randomly.

As was recommended, we estimated that a sample of 470 students would be enough for this study (10 individuals per item of the questionnaire) [31]. However, 717 students of 31 classes participated in the study.

The study objectives were explained to the students, and after obtaining informed consent, participants completed the HELMA. Of 717 returned questionnaires, 65 were removed as the respondents’ ages were outside of the age range 15–18 years. 60 of the returned questionnaires were excluded as they had data missing. Statistical analysis was carried out using the remaining complete data. Demographic characteristics of the study participants, such as age, gender, grade, parents’ jobs and education, first source of health information, and overall health status, were also recorded.

#### ii) Statistical analysis

The psychometric properties of HELMA were examined with several statistical analyses as follows:

1. *Construct validity*: To investigate the construct validity, exploratory factor analysis with varimax rotation was used. To determine the number of required factors, the analysis was carried out multiple times using a different number of factors. To determine the number of potential underlying factors, eigenvalues above 1 and a scree plot were used [32]. Factor loadings greater than or equal to 0.40 were considered appropriate. To assess the appropriateness of the sample for the factor analysis, The Kaiser-Meyer-Olkin (KMO) and Bartlett’s Test of Sphericity were used [32,33].
2. *Reliability*: Internal consistency and test-retest analyses were used in order to determine the reliability of the HELMA. To determine the internal consistency, the Cronbach’s (alpha) coefficient was measured for each dimension as well as for the entire scale. A Cronbach’s (alpha) coefficient of 0.70 or above indicates that the instrument has acceptable reliability [34]. To examine the instrument’s stability, test-retest was used. Two samples of adolescents (22 boys and 22 girls) completed the HELMA twice with a two-week interval [31,35], and the Intraclass Correlation Coefficients (ICC) were calculated where a value of 0.4 or above was considered to be acceptable [35]. All of the statistical analyses were performed using SPSS, version 17.0.

### Ethics statement

The ethics committee of the Shahid Beheshti University of Medical Sciences approved the study. In addition to this, we obtained permission from authorities in the Ministry of Education. We also obtained verbal informed consent from all of the study’s participants. The consent was obtained verbally as we thought students may otherwise feel uneasy. In doing so, the main investigator (SHGH) introduced herself and the study’s objectives. The participants were then either interviewed (Phase 1) or given the questionnaire to complete (Phase 2) in private. They were ensured that their name and information would be kept confidential and they were told that they were free to leave the interview or not complete the study questionnaire if they did not wish to do so. This is considered to be an acceptable consent procedure by the ethics committees both at institutional level (Shahid Beheshti University of Medical Sciences) and at national level (Ministry of Health) for exceptional situations, and was confirmed for this study in particular.

## Results

### The sample characteristics

In all, 582 adolescents aged 15–18 participated in the study. The mean age of respondents was 16.2 years, 51.2% were females, 44.2% were interested or very interested in health issues, and 75.7% rated their overall health status as “good” or “very good”. 44.2% of respondents said that their parents were their first source of health information and a much lower percentage of 29.4%, claimed that the Internet was their first source of health information (Table 1).

**Table 1:** Demographic characteristics of adolescents participated in study (n = 582).

	No. (%)
**Age (mean, SD**^a^**)**	16.2(1.03)
**Gender**	
Female	298 (51.2)
Male	284 (48.8)
**High school Grade**	
First	175 (30.1%)
Second	181 (31.1%)
Third	146 (25.1%)
Forth	80 (13.7%)
**Mother education**	
Illiterate	12 (2.1%)
Primary	126 (21.8%)
Secondary	326 (56.4%)
Higher	114 (19.7%)
**Father education**	
Illiterate	5 (0.9%)
Primary	106 (18.7%)
Secondary	279 (49.1%)
Higher	178 (31.3%)
**Mother job**	
Housewife	455 (82.3%)
Employed	98 (17.7%)
**Father job**	
Employed	488 (86.2%)
Unemployed	18 (3.2%)
Retired	60 (10.6%)
**Interested in health issues**	
Not interested	38 (6.6%)
Little	68 (11.7%)
Somewhat	228 (39.3%)
Much	149 (25.7%)
Very much	97 (16.7%)
**Health Status**	
Very good	179 (30.8%)
Good	261 (44.9%)
Fair	106 (18.2%)
Poor	26 (4.5%)
Very poor	9 (1.5%)
**Source of health information**	
Teacher	25 (4.3%)
Parents	257 (44.2%)
Physician	80 (13.8%)
Internet	171 (29.4%)
Health provider	2 (0.4%)
Friends, Relatives	27 (4.6%)
Book	19 (3.3%)

a. SD = Standard deviation.

### Factor structure

After confirming the adequacy of the sampling based on the KMO and Bartlett’s Test of Sphericity (KMO = 0.917 and χ^2^ = 11239.38, p< 0.0001), 11 factors emerged with eigenvalues of greater than 1, which accounted for 60.50% of the variance observed. Factor loading patterns of the 6 areas of reading, understanding, appraisal, communication and numeracy were consistent with the initial assumed item set. Due to the low loading of some factors, and in accordance with the dimensions of health literacy, the number of factors was limited to 8, with minimum factor loading of 0.45. Finally, 44 items were loaded in 8 factors that explained 53.37% of the variance, representing the following dimensions: access (5 items), reading (5 items), understanding (10 items), appraisal (5 items), use (4 items), communication (8 items), self-efficacy (4 items), and numeracy (3 items). The results are shown in Table 2.

**Table 2:** The result obtained from exploratory factor analysis with varimax rotation among adolescents aged 15–18 (n = 582).

Items	Factor1	Factor2	Factor3	Factor4	Factor5	Factor6	Factor7	Factor8
1. I try to get more information about health as much as possible	.109	.149	.201	.062	.011	.458	**.521**	-.099
2. I am able to find more information on health	.130	.110	.130	.111	.274	.044	**.575**	.039
3. When ill or facing health problems, I can get the necessary information I need	.057	.126	.141	.080	.310	-.035	**.578**	.002
4. I am able to ask others about health information that I need	.203	.290	.004	-.016	.160	-.088	**.499**	.080
5. I am able to access information about the healthy diet that is appropriate for my age group	.098	.082	-.029	.059	**.589**	.260	.289	.097
6. I am able to access information about the physical activities appropriate for my age group	.148	.067	-.187	.059	**.549**	.388	.151	.031
7. I am able to access information about the proper care required for my skin and hair that is appropriate for my age group	.027	.047	-.030	.078	**.634**	.092	.186	-.060
8. I am able to access information about mental health appropriate for my age group	.082	.164	.154	.126	**.598**	.022	.177	-.043
9. I am able to find useful resources about health information on the internet	.074	.208	.275	.063	**.663**	-.138	-.095	-.065
10. I can read brochures on prescribed medicine	.229	.092	**.717**	.125	.017	.117	.170	-.069
11. I can easily read educational brochures about nutritional issues	.295	.088	**.732**	.113	.121	.176	.098	-.023
12. I can easily read brochures/fact sheets about disease prevention (e.g. anemia, osteoporosis, respiratory infections, etc.)	.329	.173	**.710**	.087	.032	.082	.149	.004
13. I can easily read health information materials in magazines and newspapers	.373	.126	**.696**	.091	.072	.121	.060	-.005
14. I can easily read health information materials on the internet (e.g. websites)	.267	.126	**.534**	.138	.439	-.050	-.120	-.035
15. I can easily understand the meaning of the signs used in hospitals and medical centres	**.488**	.093	.301	.238	.140	.091	.005	.012
16. I can understand most things I hear about health	**.646**	.156	.129	.238	.034	.080	.183	-.154
17. I can easily understand the content of health information that I find	**.612**	.171	.188	.209	.083	.070	.165	-.018
18. I can easily understand my doctor’s instructions and recommendations (e.g. prescriptions)	**.676**	.173	.046	.141	.039	.008	.236	-.189
19. I can easily understand information about medications–usage, side effects and warnings	**.650**	.181	.146	.199	.001	-.018	.242	-.092
20. I can easily understand the nutritional facts on prepared food packages	**.601**	-.010	.163	.277	.140	.133	-.009	.003
21. I can understand the information and recommendations about proper nutrition for adolescents in the media (e.g. radio, TV, internet, etc.)	**.668**	.148	.185	.137	.187	.132	-.027	-.088
22. I can understand the information and warnings provided by the media (e.g. radio, TV, internet, etc.) about tobacco, drug abuse and risky behavior	**.683**	.167	.199	-.097	.074	.184	-.145	-.006
23. I can understand the information and recommendations about health and illness in the media	**.695**	.204	.195	.085	.104	.085	.058	.020
24. I can understand the recommendations on prevention of accidents and injuries	**.556**	.219	.262	.205	.022	-.024	.114	-.031
25. When faced with new health information, I can judge its accuracy	.272	.103	.064	**.596**	.102	.062	.198	-.038
26. I would compare the data obtained from various sources	.175	.215	.139	**.671**	-.012	.187	.066	.016
27. When dealing with conflicting information about health issues, I can recognize the correct information	.260	.203	.058	**.724**	.103	.060	-.008	.027
28. I have the ability to judge which resources I can trust	.182	.221	.060	**.674**	.145	.044	.058	.053
29. When dealing with nutritional information I can choose the right information	.392	.193	.148	**.559**	.143	.190	-.033	-.001
30. When shopping, I choose food based on its nutritional facts (e.g. amount of energy, sugar, fats, protein, etc.) written on the packaging	.060	.003	.185	.426	.089	**.542**	-.038	.117
31. I try to choose foods without preservatives	.018	.129	.151	.248	.095	**.645**	-.017	.006
32. I try to apply what I have learned about health issues in my everyday life	.206	.272	.117	.209	.020	**.617**	.154	-.108
33. I try to keep my body weight in balance	.166	.224	-.024	-.139	.077	**.529**	.002	.082
34. I can discuss my concerns relating to health issues with health providers	-0.41	**.521**	.175	.199	.215	.305	-.046	.020
35. When visiting a doctor or health provider I am able to give him/her all of my necessary personal information	.233	**.586**	-.040	.109	.117	.117	.174	-.022
36. When visiting a doctor or health provider I am able to tell him/her the name of the medications that I have previously used	.089	**.549**	.093	.180	.116	.091	.055	.001
37. When visiting a doctor or health provider I am able to ask all the questions I have	.233	**.667**	-.030	.084	.195	.118	.021	.043
38. I can share the health information that I gather with others (e.g. family, friends, etc.)	.254	**.578**	.100	.124	-.057	.092	.203	-.156
39. If I have any questions about health issues, I am able to get information and advice from others	.123	**.670**	.126	.176	.129	.035	.181	-.097
40. When visiting a doctor or health provider, I am able to ask questions based on my research	.098	**.710**	.211	.230	.173	.021	.131	-.038
41. I talk to my friends about avoiding risky behaviour (e.g. smoking, hookah, drugs, etc.)	.138	**.524**	.063	.010	-.017	.150	.058	.044
42. If a person drinks 3 cups of milk in one given day, how many carbohydrates has s/he recieved? (based on the given information in questionnare)	-.108	.003	.012	.033	-.083	.036	.107	**.586**
43. Calculate the BMI of a person with height = 160 cm and weight = 70 kg?	-.078	-.061	-.046	.029	-.067	-.053	-.051	**.884**
44. What is this person’s BMI status? (based on the given information in questionnaire)	-.055	-.056	-.039	.003	.094	.017	-.038	**.758**
**Eigenvalue**	**12.26**	**2.72**	**2.17**	**2.08**	**1.68**	**1.54**	**1.39**	**1.26**
**Explained Variance (%)**	**26.08**	**5.78**	**4.61**	**4.42**	**3.57**	**3.28**	**2.95**	**2.67**
**Cumulative Variance (%)**	**26.08**	**31.86**	**36.47**	**40.89**	**44.47**	**47.75**	**50.70**	**53.37**

Factors: 1 = understanding, 2 = communication, 3 = reading, 4 = appraisal, 5 = access, 6 = use, 7 = self-efficacy and 8 = numeracy.

### Reliability

The Cronbach’s (alpha) coefficient for the entire scale was 0.93 and ranged from 0.61 to 0.89 for various domains. The intraclass correlation coefficient (ICC = 0.93) also indicated that HELMA had satisfactory stability (Table 3).

**Table 3:** Cronbach’s α coefficient and ICC for the HELMA and its subscales.

Domain	Number of items	Cronbach’s α coefficient (n = 582)	ICC^a^ (n = 44)
**Self-efficacy**	4	0.61	0.79
**Access**	5	0.71	0.62
**Reading**	5	0.86	0.88
**Understanding**	10	0.89	0.84
**Appraisal**	5	0.81	0.75
**Use**	4	0.65	0.86
**Communication**	8	0.83	0.77
**Numeracy**	3	0.65	0.60
**Total scale**	44	0.93	0.93

a. ICC = intraclass correlation coefficient.

## Discussion

This study was the first attempt to design and psychometrically evaluate an instrument to measure health literacy among adolescents in Iran. The initial questionnaire was developed based on data obtained from a qualitative study on adolescents aged 15–18, expert opinions, and extensive reviews of existing literature on health literacy. The designed questionnaire included a wide range of items to assess individual and interpersonal factors relating to adolescent health literacy. After the completion of the validity and reliability phases, the health literacy measure for adolescents (HELMA) consisted of 44 items within 8 areas. These areas were access, reading, understanding, appraisal, use, communication, self-efficacy and numeracy. As most participants completed the questionnaire without any difficulties in approximately 15 minutes, we believe that the HELMA is an easy-to-use questionnaire that can be used easily for future studies.

Some existing studies in Iran are limited as they must use translated versions of the better known instruments, such as the Test of Functional Health Literacy in Adults (TOFHLA) and the Newest Vital Sign (NVS) [36–42].

Over the past two years, two new health literacy questionnaires were developed, one specifically for oral health and one for the adult urban population aged 18–65 [43,44], however, adolescent health literacy was not addressed.

Measuring health literacy among adolescents, not only in Iran but also elsewhere, is a relatively new issue. Although there are growing concerns about this, applying conventional health literacy measurements (such as TOFHLA, REALM, NVS) to this population has its limitations [45]. Few studies have investigated adolescent health literacy and fewer research projects have addressed this concept from adolescents’ perspectives.

The findings of this study indicated that the HELMA has appropriate validity and reliability. One of the features of the HELMA is that, in addition to functional health literacy, it captures other dimensions including interactive health literacy and critical health literacy. Given that health literacy is a context-specific concept [20], studying adolescents’ health literacy in a school is another important aspect of this study, which can help to overcome the challenges of health literacy assessments in non-clinical environments, particularly since this age group is often in the healthiest period of life and have less dealings with healthcare services [21].

Studies suggest that health literacy is a social feature that should be considered as a latent structure with multiple dimensions. The complex nature of the health literacy notion confirms the necessity to use a multi-dimensional tool [13,46]. The factor structure analysis of the few tools designed to assess adolescents' health literacy showed different patterns. For instance, Massey et al. introduced a multi-dimensional tool that captured health information seeking items in 3 distinct factors: health information seeking skills, confidence in health information from human sources and confidence in health information from media sources [13].

Abel et al. [20] developed a 4-dimension instrument that assesses functional, interactive and critical health literacy in adolescents. In this scale, the functional health literacy domain was reported in two discrete factors: the finding of health information and the understanding of health information.

In comparison to the mentioned studies, the factor structure of the HELMA is more varied, and due to each factor having a minimum of three items, the HELMA has a more reliable construction [47]. During the development of the items, we used the competencies necessary to address everyday life health issues that, in addition to capturing different aspects of health literacy, increased the validity of the HELMA. In the factor analysis, the items designed for the “access” area were loaded into two separate factors. In other words, to obtain health information as well as the ability to gain access to health information sources, self-confidence in attaining and using these resources was necessary.

Despite the large information gap between two generations, parents were the primary source of health information for almost half of the adolescents. The Internet was said to be the primary source of 29.4% of the respondents.

Based on the health literacy survey conducted in 2007 in five provinces, including Tehran, only 28.1% of adults have adequate health literacy [36]. Low health literacy of parents and wide and often unsupervised access of adolescents to the Internet, endorsed the need for educational interventions to improve the health literacy skills of this age group. Among the various sources of health information, physicians and health providers were in third place, which is most likely because adolescents are generally healthy and consequently have fewer interactions with the healthcare system.

As stated previously, this was the first attempt to measure the health literacy of Iranian adolescents. Future studies should be carried out among different age goups of adoelscents and in different environments. Perhaps the evaluation of such studies may lead to a stronger confirmation of the psychometric properties of the HELMA. For instance we feel that the variance obseved in the exploratory factor analysis was relatively low and this might increase in different settings. At present, both Persian and English versions of the quesionnaire are available.

## Conclusion

The Health Literacy Measure for Adolescents (HELMA) is a valid and reliable instrument to measure the health literacy of adolescents and can be used for future studies.

## Supporting Information

S1 File The Health Literacy Measure for Adolescents (HELMA)-English version. (DOC)

S2 File The Health Literacy Measure for Adolescents (HELMA)-Persian version. (DOC)

S3 File A minimal set of data for the study. (SAV)

S4 File Scoring for the HELMA. (DOC)
