# Supplementary material for: Health Literacy Measure for Adolescents (HELMA): Development and Psychometric Properties
Source: PLoS One. 2016 Feb 16;11(2):e0149202. doi: 10.1371/journal.pone.0149202 (PMC4755574; doi:10.1371/journal.pone.0149202)
Supplement: S1 File — (DOC) [file pone.0149202.s001.doc]

**Health Literacy Measure for** **Adolescents (HELMA)**

Dear respondent,

This questionnaire deals with your perceived abilities and competencies on obtaining and using health information. For each question, put a check mark or cross in the box in front of the answer that best describes your abilities or skills. Please answer all questions.

| **Items** | **Never** | **Rarely** | **Sometimes** | **Usually** | **Always** |
| --- | --- | --- | --- | --- | --- |
| 1. I try to get more information about health as much as possible |  |  |  |  |  |
| 2. I am able to find health information that I need |  |  |  |  |  |
| 3. When ill or facing health problems, I can get the necessary information I need |  |  |  |  |  |
| 4. I am able to ask others about health information that I need |  |  |  |  |  |
| 5. I am able to access information about the healthy diet that is appropriate for my age group |  |  |  |  |  |
| 6. I am able to access information about the physical activity appropriate for my age group |  |  |  |  |  |
| 7. I am able to access information about the proper care required for my skin and hair that is appropriate for my age group |  |  |  |  |  |
| 8. I am able to access information about mental health appropriate for my age group |  |  |  |  |  |
| 9. I am able to find useful resources about health Information on the Internet |  |  |  |  |  |
| 10. I can read brochures on prescribed medicine |  |  |  |  |  |
| 11. I can easily read educational brochures about nutritional issues |  |  |  |  |  |
| 12. I can easily read brochures/fact sheets about disease prevention (e.g. anaemia, osteoporosis, respiratory infections, etc.) |  |  |  |  |  |
| 13. I can easily read health information materials in magazines and newspapers |  |  |  |  |  |
| 14. I can easily read health information materials on the Internet (e.g. websites) |  |  |  |  |  |

| **Items** | **Never** | **Rarely** | **Sometimes** | **Usually** | **Always** |
| --- | --- | --- | --- | --- | --- |
| 15. I can easily understand the meaning of the signs used in hospitals and medical centres |  |  |  |  |  |
| 16. I can understand most things I hear about health |  |  |  |  |  |
| 17. I can easily understand the content of health information that I find |  |  |  |  |  |
| 18. I can easily understand my doctor’s instructions and recommendations (e.g. prescriptions) |  |  |  |  |  |
| 19. I can easily understand information about medications – usage, side effects and warnings |  |  |  |  |  |
| 20. I can easily understand the nutrition facts on food packages |  |  |  |  |  |
| 21. I can understand the information and recommendations about proper nutrition for adolescents in the media (e.g. radio, TV, internet, etc.) |  |  |  |  |  |
| 22. I can understand the information and warnings provided by the media (e.g. radio, TV, internet, etc.) about tobacco, drug abuse and risky behaviours |  |  |  |  |  |
| 23. I can understand the information and recommendations about health and illness in the media |  |  |  |  |  |
| 24. I can understand the recommendations on prevention of accidents and injuries |  |  |  |  |  |
| 25. When faced with new health information, I can judge its accuracy |  |  |  |  |  |
| 26. I would compare the data obtained from various sources |  |  |  |  |  |
| 27. When dealing with conflicting information about health issues, I can recognize the correct information |  |  |  |  |  |
| 28. I have the ability to judge which resources I can trust |  |  |  |  |  |
| 29. When dealing with nutritional information I can choose the right information |  |  |  |  |  |

| **Items** | **Never** | **Rarely** | **Sometimes** | **Usually** | **Always** |
| --- | --- | --- | --- | --- | --- |
| 30. When shopping, I choose food based on its nutrition facts (e.g. amount of energy, sugar, protein, etc.) written on the packaging |  |  |  |  |  |
| 31. I try to choose foods without preservatives |  |  |  |  |  |
| 32. I try to apply what I have learned about health issues in my everyday life |  |  |  |  |  |
| 33. I try to keep my body weight in balance |  |  |  |  |  |
| 34. I can discuss my concerns relating to health issues with health providers |  |  |  |  |  |
| 35. When visiting a doctor or health provider I am able to give him/her all of my necessary personal information |  |  |  |  |  |
| 36. When visiting a doctor or health provider I am able to tell him/her the name of the medications that I have previously used |  |  |  |  |  |
| 37. When visiting a doctor or health provider I am able to ask all the questions I have |  |  |  |  |  |
| 38. I can share the health information that I gather with others (e.g. family, friends, etc.) |  |  |  |  |  |
| 39. If I have any questions about health issues I am able to get information and advice from others |  |  |  |  |  |
| 40. When visiting a doctor or health provide I am able to ask questions based on my research |  |  |  |  |  |
| 41. I talk to my friends about avoiding risky behaviour (e.g. smoking, hookah, drugs, etc.) |  |  |  |  |  |

42. This information is on the back of a container of milk. If a person drinks 3 cups of milk in one given day, how many carbohydrates has he/she received?

| **Nutrition facts** |
| --- |
| **Serving size:** 1 cup (240 cc) |
| **Servings per container**: 4 |
| **Amount per serving:** |
| **Energy**: 140 Kcal |
| **Total Fat:** 7gr  **Cholesterol:** 30 mg |
| **Carbohydrates:** 11gr  **Sugar:** 0 gr |
| **Protein**: 8gr |
| **Sodium:** 160 mg |

43. Calculate the BMI of a person with height=160 cm and weight=70 kg?

BMI=
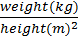


44. What is this person’s body fat status (based on the following information)?

|  | **Underweight** | **Normal weight** | **Overweight** | **Obese** |
| --- | --- | --- | --- | --- |
| **BMI** | **<18.5** | **18.5-24.9** | **25-29.9** | **≥30** |

a-Underweight b- Normal weight

c- Overweight d- Obese

**Thank you for completing the questionnaire**

**©** Ghanbari Sh. et al., 2015
