# Supplementary material for: Health Literacy Measure for Adolescents (HELMA): Development and Psychometric Properties
Source: PLoS One. 2016 Feb 16;11(2):e0149202. doi: 10.1371/journal.pone.0149202 (PMC4755574; doi:10.1371/journal.pone.0149202)
Supplement: S2 File — (DOC) [file pone.0149202.s002.doc]

**دانش آموز عزیز**

پرسشنامه حاضر مربوط به یک طرح علمی و پژوهشی در رابطه با بررسی توانایی و مهارت های نوجوانان در رابطه با کسب و استفاده از اطلاعات سلامت است. لطفا پاسخ خود را در جلو هر سوال با علامت Х یا 🗸 در ستون مورد نظر مشخص نمایید. اطلاعات پرسشنامه ها بدون نام و اطلاعات آن محرمانه می باشد. لطفا به تمام سوالات پاسخ دهید.

| **ردیف** | **سوال** | **هرگز** | **به ندرت** | **بعضی اوقات** | **بیشتر اوقات** | **همیشه** |
| --- | --- | --- | --- | --- | --- | --- |
| 1 | سعی می ‌کنم تا حد امکان اطلاعات بیشتری در مورد سلامت کسب کنم |  |  |  |  |  |
| 2 | مهارت لازم برای پیدا کردن اطلاعات بهداشتی مورد نیازم را دارم |  |  |  |  |  |
| 3 | می توانم در هنگام مواجهه با بیماری یا مشکلات تهدید کننده سلامتی اطلاعات لازم را به دست آورم |  |  |  |  |  |
| 4 | می توانم در مورد اطلاعات بهداشتی مورد نیازم از دیگران سوال کنم |  |  |  |  |  |
| 5 | می توانم اطلاعات مربوط به روش تغذیه سالم متناسب با سن خودم را به دست آورم |  |  |  |  |  |
| 6 | می توانم اطلاعات لازم در مورد روش ورزش صحیح متناسب با سن خودم را به دست آورم |  |  |  |  |  |
| 7 | می توانم اطلاعات لازم در مورد روش صحیح مراقبت از پوست و مو متناسب با سن خودم را به دست آورم |  |  |  |  |  |
| 8 | می توانم اطلاعات مورد نیاز در زمینه بهداشت روانی متناسب با سن خودم را به دست آورم |  |  |  |  |  |
| 9 | می توانم منابع مفید برای اطلاعات سلامت را در اینترنت پیدا کنم |  |  |  |  |  |
| 10 | می توانم بروشورهای فارسی داخل جعبه های دارو را بخوانم |  |  |  |  |  |
| 11 | می توانم برگه های آموزشی در مورد موضوعات مربوط به تغذیه را به راحتی بخوانم |  |  |  |  |  |
| 12 | می توانم بروشورها یا برگه های اطلاعاتی در مورد پیشگیری از بیماری ها (مثل کم خونی، پوکی استخوان، عفونت های تنفسی و .. ) را به راحتی بخوانم |  |  |  |  |  |
| 13 | می توانم مطالب بهداشتی موجود در مجلات و روزنامه ها را به راحتی بخوانم |  |  |  |  |  |
| 14 | می توانم مطالب بهداشتی موجود در سایت ها را به راحتی بخوانم |  |  |  |  |  |
| 15 | می توانم مفهوم تابلوهای راهنما و علائم موجود در بیمارستان ها و مراکز درمانی را به راحتی درک کنم |  |  |  |  |  |
| 16 | می توانم اکثر چیزهایی را که در مورد سلامت می شنوم، درک کنم |  |  |  |  |  |
| **17** | می توانم محتوای اطلاعات بهداشتی را که پیدا کرده ام به راحتی درک کنم |  |  |  |  |  |
| 18 | می توانم دستورات و توصیه های پزشک را به راحتی درک کنم |  |  |  |  |  |
| 19 | می توانم اطلاعات مربوط به نحوه مصرف، عوارض و هشدارهای دارویی را به راحتی درک کنم |  |  |  |  |  |
| 20 | می توانم اطلاعات جداول تغذیه ای روی بسته های مواد غذایی آماده را به راحتی درک کنم |  |  |  |  |  |
| 21 | می توانم اطلاعات و توصیه های مربوط به تغذیه مناسب برای نوجوانان در رسانه ها ( رادیو، تلویزیون، اینترنت و ... ) را درک کنم |  |  |  |  |  |
| 22 | می توانم اطلاعات و هشدارهای ارائه شده در رسانه ها ( رادیو، تلویزیون، اینترنت و ... ) در مورد مصرف دخانیات، مواد مخدر و یا سایر رفتار های پر خطر را درک کنم |  |  |  |  |  |
| 23 | می توانم اطلاعات و توصیه های مربوط به سلامت و بیماری در رسانه ها ( رادیو، تلویزیون، اینترنت و ... ) را درک کنم |  |  |  |  |  |
| 24 | می توانم توصیه های مربوط به اقدامات پیشگیری از سوانح و حوادث را درک کنم |  |  |  |  |  |
| 25 | وقتی با اطلاعات جدید در مورد سلامت رو به رو می شوم می توانم در مورد صحت آنها قضاوت کنم |  |  |  |  |  |
| 26 | من اطلاعات به دست آمده از منابع مختلف را با هم مقایسه می کنم |  |  |  |  |  |
| 27 | در صورت برخورد با اطلاعات متناقض در زمینه موضوعات بهداشتی، می‌توانم اطلاعات صحیح را تشخیص دهم |  |  |  |  |  |
| 28 | مهارت لازم برای قضاوت در مورد اینکه به کدام منبع اطلاعاتی می توانم اعتماد کنم را دارم |  |  |  |  |  |
| 29 | در هنگام برخورد با اطلاعات تغذیه ای می توانم اطلاعات صحیح را انتخاب کنم |  |  |  |  |  |
| 30 | به هنگام خرید با توجه به جدول ارزش غذایی( میزان انرژی، میزان قند، چربی، پروتئین و ...) نوشته شده بر روی بسته های مواد غذایی، آنها را انتخاب می کنم |  |  |  |  |  |
| 31 | سعی می کنم مواد غذایی را انتخاب کنم که مواد نگه دارنده نداشته باشند |  |  |  |  |  |
| 32 | سعی می کنم آنچه که در مورد موضوعات مربوط به سلامت یاد گرفته ام را در زندگی روزمره اجرا کنم |  |  |  |  |  |
| 33 | برای متعادل نگه داشتن وزن بدنم تلاش می کنم |  |  |  |  |  |
| 34 | می توانم در باره نگرانی هایم در مورد موضوعات مربوط به سلامت با کارکنان بهداشتی صحبت کنم |  |  |  |  |  |
| 35 | در هنگام مراجعه به پزشک یا کارمند بهداشتی می توانم همه اطلاعات لازم در مورد وضعیت خودم را به آنها بدهم |  |  |  |  |  |
| 36 | در هنگام مراجعه به پزشک یا کارمند بهداشتی می توانم نام داروهایی که قبلا استفاده کرده ام را به آنها بدهم |  |  |  |  |  |
| 37 | در هنگام مراجعه به پزشک یا کارمند بهداشتی، می توانم تمام سوالات مورد نیازم را بپرسم |  |  |  |  |  |
| 38 | می توانم اطلاعات بهداشتی که به دست آورده ام را به دیگران (دوست، افراد خانواده ... ) منتقل کنم |  |  |  |  |  |
| 39 | اگر سوالی در مورد موضوعات بهداشتی داشته باشم می توانم از دیگران اطلاعات و مشاوره دریافت کنم |  |  |  |  |  |
| 40 | در هنگام مراجعه به پزشک یا کارمند بهداشتی می توانم با توجه به تحقیقات و اطلاعاتی که به دست آورده ام، سوالات خودم را مطرح کنم |  |  |  |  |  |
| 41 | در رابطه با اجتناب از رفتار های مخاطره آمیز ( کشیدن سیگار، قلیان، مواد مخدر، دوستی های خیابانی و ...) با دوستانم صحبت می کنم |  |  |  |  |  |

42- بر روی پاکت یک نوع شیر اطلاعات زیر درج شده است. اگر فردی در روز سه لیوان شیر بخورد چقدر کربوهیدرات دریافت کرده است؟...........................

| **جدول ارزش غذایی** |
| --- |
| **اندازه یک سهم:** یک لیوان (240 سی سی) |
| **حجم کل:** 1000 سی سی ( یک لیتر) |
| **مقادیر مربوط به یک سهم** |
| **انرژی:** 140 (کیلو کالری) |
| **چربی کل:** 7 گرم  کلسترول: 30 میلی گرم |
| **کربوهیدرات:** 11 گرم  شکر: صفر گرم |
| **پروتئین:** 8 گرم |
| **سدیم:** 160 میلی گرم |

متخصصین تغذیه برای ارزیابی وضعیت چاقی یا لاغری افراد از شاخصی به نام شاخص توده بدنی استفاده می کنند برای محاسبه این شاخص از فرمول زیر استفاده می کنند و نتیجه را بر اساس جدول طبقه بندی می کنند

**وزن به کیلو گرم  = شاخص توده بدنی**

**( قد به متر )^2^**

| شاخص توده بدنی | کمتر از 5/18 | 9/24 -5/18 | 9/29 – 0/25 | بیشتر از 0/30 |
| --- | --- | --- | --- | --- |
| طبقه بندی وزن | لاغر | طبیعی | دارای اضافه وزن | چاق |

43- اگر قد فردی 160 سانتی متر (یک متر و شصت سانتی متر) و وزنش70 کیلوگرم باشد،شاخص توده بدنی این فرد چقدر است؟................................

44- بر اساس شاخص توده بدنی وزن این فرد در کدام محدوده قرار می گیرد:

الف_ لاغر🗌 ب- طبیعی 🗌 ج- دارای اضافه وزن 🗌 د- چاق 🗌

©1394. تمامی حقوق مادی و معنوی این پرسشنامه متعلق به شهلا قنبری و همکاران می باشد.
