# Supplementary material for: Health Literacy Measure for Adolescents (HELMA): Development and Psychometric Properties
Source: PLoS One. 2016 Feb 16;11(2):e0149202. doi: 10.1371/journal.pone.0149202 (PMC4755574; doi:10.1371/journal.pone.0149202)
Supplement: S4 File — (DOC) [file pone.0149202.s004.doc]

**Manual for scoring the HELMA**

|  | **Number of items** | **Minimum possible raw score** | **Maximum possible raw score** |
| --- | --- | --- | --- |
| **Access** | **5 (item 5-9)** | **5** | **25** |
| **Reading** | **5 (item 10-14)** | **5** | **25** |
| **Understanding** | **10 (item 15-24)** | **10** | **50** |
| **Appraisal** | **5 (item 25-29)** | **5** | **25** |
| **Use** | **4 (item 30-33)** | **4** | **20** |
| **Communication** | **8 (item 34-41)** | **8** | **40** |
| **Self-efficacy** | **4 (item 1-4)** | **4** | **20** |
| **Numeracy** | **3 (item 42-44)** | **3** | **15** |

To calculate each subscale or total score for the HELMA, first we added raw scores and linearly transferred it to a score from 0 to 100 using the following formula.

**Score=**
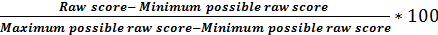


We ranked the HELMA score to 4 categories: ‘inadequate’, ‘problematic’ (which together also define ‘limited’ health literacy), ‘sufficient and ‘excellent’ (which together also defined ‘desired’ health literacy):

Inadequate= 0–50

Problematic= 50.1–66

Sufficient= 66.1–84

Excellent=84.1-100

**©** Ghanbari Sh. et al., 2015
